# Risk of spontaneous preterm labor in pregnancies achieved by in vitro fertilization and complicated with severe form of ovarian hyperstimulation syndrome: A case control study

**DOI:** 10.12669/pjms.35.4.145

**Published:** 2019

**Authors:** Aleksandar Dobrosavljevic, Snezana Rakic, Sladjana Mihajlovic

**Affiliations:** 1 Dr. Aleksandar Dobrosavljevic, Obstetrician and Gynecologist, The Obstetrics and Gynecology Clinic Narodni Front, Kraljice Natalije 62, 11000 Belgrade, Serbia; 2 Dr. Snezana Rakic, Professor of Obstetrics and Gynaecology, The Obstetrics and Gynecology Clinic Narodni Front, Kraljice Natalije 62, 11000 Belgrade, Serbia. University of Belgrade School of Medicine, Dr Subotica 8, Serbia; 3 Sladjana Mihajlovic, Assistant Professor of Obstetrics and Gynaecology, The Obstetrics and Gynecology Clinic Narodni Front, Kraljice Natalije 62, 11000 Belgrade, Serbia. University of Belgrade School of Medicine, Dr Subotica 8, Serbia

**Keywords:** IVF/ICSI, Ovarian hyper stimulation syndrome, Preterm birth

## Abstract

**Objectives:**

The purpose of this study was to examine the potential impact of severe Ovarian hyper stimulation syndrome (OHSS) on the risk of preterm birth. Severe ovarian hyperstimulation syndrome is a serious complication in the methods of in vitro fertilization. The pathophysiology of this process is not clear enough and the treatment is symptomatic. Human chorionic gonadotropin (h-CG) is the most important known cause of this condition. Findings of other authors often do not match when it comes to complications that may occur in pregnancy.

**Methods:**

In the Gynecology and Obstetrics Clinic “Narodni Front” a case control study was conducted on 50 female patients with severe forms of OHSS in the period from January 2008 to March 2015. A control group was created based on age and it involved 59 patients with pregnancy achieved with IVF/ICSI during the same period, but in which OHSS did not occur.

**Results:**

Patients with the pregnancy complicated by OHSS, had a considerably higher rate of preterm labor, whether this was labor before gestation week 37 (56.0% vs. 30.5%) or before gestation week 34 (34.0% vs. 6.8%); significantly lower weight of newborns, as in the newborns with low body weight <2500g (45.6% vs. 25.0%) and specially in the newborn with very low body weight <1500 grams (19.1% vs. 3.8%), as well as preterm premature rupture of membranes (PPROM), (11.76% vs. 1.59%).

**Conclusions:**

Pregnancy achieved by the IVF/ICSI method in which severe form of OHSS has been developed could have an increased risk of preterm birth.

## INTRODUCTION

OHSS is one of the most serious and sometimes life-threatening complications in the methods of in vitro fertilization. The severe form of OHSS occurs with an incidence of 0.5 to 5%.^1^ It is considered that the most important factor for the development of this, often life-threatening condition, is hCG.^2^ It can be divided into early and late. Early occurs shortly after oocyte aspiration and is the result of the influence of exogenous hCG that is administered for the purpose of final oocyte maturation. The predisposing factors for its development are: younger age, PCOS, tubal and male sterility. The late OHSS form occurs ten or more days after administration of hCG and occurs as a result of endogenous production of hCG. The late form has a tendency to be more severe and more difficult to predict.^3^ An increased secretion of mediators by granulosa and lutein cells, among which a special place occupies a vascular endothelial growth factor (VEGF), leads to an increased permeability of the capillaries and the accumulation of fluid in the so-called third space. Further course leads to the formation of ascites and/or pleural effusion. The loss of fluid from the vascular compartment leads to hemoconcentration reduced renal perfusion followed by oliguria, renal insufficiency and thrombosis. In addition to VEGF as the dominant mediator, the soluble receptor sFlt-1 and interleukins also play an important role.^4^ In accordance with the fact that the pathophysiological process is not sufficiently defined; there is no specific treatment, so the therapy is primarily symptomatic.^5^

The results of the studies that deal with the effects of severe OHSS on the outcome of pregnancies are controversial.^6^,^7^ The purpose of this study was to examine the risk of preterm birth in female patients with the severe form of OHSS.

## METHODS

In the Gynecology and Obstetrics Clinic “Narodni Front” a case control study was conducted on 50 female patients with severe forms of OHSS in the period from January 2008 to March 2015. The study was approved by the Institutional Research Ethics Board (record number 24/13-3). The study was conducted in accordance with the Helsinki Declaration and other nationally valid regulations. The control group consisted of 59 patients in whom there has been no development of OHSS and who achieved their pregnancy by IVF during the same time period and who were of appropriate age. All pregnant women included in the study were nulliparous. The definition of the severe OHSS form is based on the criteria of Golan and associates (1989) and Navot and associates (1992): pain in the abdomen, ovaries greater than 5cm and ascites or hydrothorax. In addition to the above mentioned clinical and/or ultrasound characteristics, patients need to fulfill one more of the following criteria: hematocrit ≥ 45%, leukocytosis> 15,000/ml, oliguria (<500ml/24h), elevated transaminases above reference, dyspnea, anasarca or acute renal insufficiency.^8^,^9^ The therapy most often involved: bed rest, application of crystalloid or colloid solution, anticoagulant therapy if indicated and drainage of ascites in certain cases. The rate of preterm birth, birth weight and preterm premature rupture of membranes (PPROM) was analyzed. In addition to the above-mentioned parameters, the following characteristics of patients were analyzed: age, cause of infertility (PCOS, anovulation, tubular factor, endometriosis, male infertility factor and idiopathic infertility), BMI, total dose of gonadotropin, protocol use with agonists or antagonists, estradiol values the day of administration of hCG as well as the number of aspirated oocytes. PCOS was defined according to the Rotterdam Consensus and involved the presence of two of the three criteria, oligo or anovulation, clinical and / or biochemical signs of hyperandrogenism and polycystic ovarian morphology.^10^ Poor respondents were considered the patients who received less than four oocytes and such patients were excluded from the study. Patients with preterm birth were first viewed as a whole, that is, all patients who had gone through labor before the 37th week of gestation, and then a group of patients who went through labor prior to the 34th week of gestation. Body weight is divided into three categories: normal body weight ≥ 2500 g.; low body weight, <2500 g (between 1500 and 2500 g); as well as very low body weight (<1500gr). IUGR is defined as fetus weight under the 10th percentile for gestational age.^11^ As it is known, this category includes numerous constitutionally small fetuses that actually do not have growth restriction, although being below the 10th percentile. Therefore, in addition to this parameter, growth dynamics and Doppler parameters were regularly monitored.

### Statistical analysis

Statistical data analysis was done in IBM SPSS program. Continuous variables are described using the lowest and highest values, mean values and standard deviations. Categorical variables are described using absolute and relative frequencies. Kolmogorov-Smirnov test was used to check the normal distribution of data. In relation to these results, for the comparison of values between the examined and the control group, the t test of independent samples is applied if the data follows the normal distribution or Mann-Whitney U test if the data does not follow the normal distribution. To search for the connection between categorical variables, a χ^2^ test is used. If any of the frequencies by categories is lower than expected, Fisher’s exact probability test would be used. The results were considered statistically significant if the p-value was less than 0.05.

## RESULTS

The rate of preterm birth was a significantly different between the study and the control group (56.0% in OHSS vs. 30.5% in control, p=0.011). (Table-I A) In the group of patients who gave birth before the 34th week of pregnancies, an even more significant difference was obtained (34.0% in OHSS vs. 6.8% in control, p=0.000).

**Table I-A:** Preterm labor (number (%)).

Preterm labor	OHSS (n=50)	Control Group (n=59)
<34	17 (34.0%)	4 (6.8%)
<37	28 (56.0%)	18 (30.5%)
>=37	22 (44.0%)	41 (69.5%)

There is statistically significant difference between preterm labor in the OHSS and control group (<34 w.g.; p=0.000); (< 37 w.g.; p=0.011). In the OHSS group we have as much as 34% of children who were born before 34th week of gestation but in the control group only 6.8%.

There is also a significant difference in preterm labor between the OHSS and control group when only singleton pregnancy is considered (40% vs 10.5%, p=0.013), but not when twin pregnancies are observed (84.2% vs 66.7%, p= 0.454). (Table-I B). The weight of newborns was significantly lower in the OHSS group, especially in newborns with very low body weight, less than 1500 grams (19.1% in OHSS vs. 3.8% in control), p=0.000. If we consider separately single fetus pregnancies there is also difference in newborns body weight between groups (20% vs 0.0%, p=0.003). In twin pregnancies, we still find a difference (18.4% vs 7.1%, p=0.002). (Table-II) Preterm premature rupture of membranes statistically significantly more often occurred in the study group (11.8% in OHSS vs. 1.6% in control, p=0.044). As for intrauterine growth restriction, there was one fetus in each group with growth restriction, which was not statistically significant. The characteristics of the patients in the study and control group are shown in Table-III A. Significant difference was obtained between the study and control group when these are viewed as a whole, for the following parameters: number of aspirated oocytes (11.93% vs 8.17%, p=0.000), estradiol values (2678.82 pg/ml vs 1702.90 pg/ml, p=0.000) total dose of gonadotropins (1759.78 vs 2325.13, p=0.000) and applied protocol (p=0.014). When single fetus pregnancies are separately observed from twin pregnancies, the results are similar. Significant difference was obtained between the number of aspirated oocytes (12.31% vs 8.3%, p<0.001), estradiol values (2692.70 pg/ml vs 1815.49, p=0.001) and total dose of gonadotropins (1869 vs 2442.95, p=0.001) (Table-III B). In twin pregnancies significant difference was obtained between: the number of aspirated oocytes (11.84% vs 7.90%, p=0.002), estradiol values (2655.43 pg/ml vs 1771.19, p≤ 0.001) total dose of gonadotropins (1817.50 vs 2532.47, p≤ 0.001) and applied protocol (p=0.010). (Table-III C) The cause of infertility and BMI were not significantly different between groups. Early form of OHSS occurred in 10 (20.0%), while the late form occurred in 40 (80%) patients. Paracentesis was performed in 12 (24.0%).

**Table I-B:** Preterm labor (number (%)) – as per pregnancy type.

Preterm labor	Singleton pregnancy		Twin pregnancy	

	OHSS (n=30)	Control group (n=38)	OHSS (n=19)	Control group (n=21)
Yes	12 (40.0%)	4 (10.5%)	16 (84.2%)	14 (66.7%)
No	18 (60.0%)	34 (89.5%)	3 (15.8%)	7 (33.3%)

There is statistically significant difference in preterm labor between the OHSS and control group when only singleton pregnancy is considered (p=0.013), but not in twin pregnancy group (p=0.454).

**Table II:** Newborns weight (mean value ± standard deviation or number (%)).

		Body weight			p

		<1500	1500-2500	>2500	
Whole group	OHSS	13 (19.1%)	31 (45.6%)	24 (35.3%)	0.000
	Control	3 (3.8%)	20 (25.0%)	57 (71.3%)	
Singleton	OHSS	6 (20.0%)	5 (16.7%)	19 (63.3%)	0.003
	Control	0 (0%)	2 (5.3%)	36 (94.7%)	
Twin	OHSS	7 (18.4%)	26 (68.4%)	5 (13.2%)	0.002
	Control	3 (7.1%)	18 (42.9%)	21 (50.0%)	

There is statistically significant difference between OHSS and control group in weight. (p=0.000) In the OHSS group we have as much as 19.1% of children below 1500 gr. but in the control group only 3.8%.

**Table III-A:** Patient’s characteristics (mean value ± standard deviation or number (%)).

	OHSS (n=50)	Control group (n=59)
Age (years)	32.47±3.92	33.60±3.54
BMI (kg/m^2^)	22.47±314	21.92±2.44
*Infertility cause*		
PCOS	14 (28.0%)	11(18.6%)
Tubal	7 (14.0%)	4(6.7%)
Endometriosis	5 (10.0%)	6(10.1%)
Anovulation	1 (2.0%)	0
Male	8 (16.0%)	19(32.2%)
Unexplained	15 (30.0%)	19(32.2%)
Number of obtained oocytes	11.93±3.48	8.17±2.453
E2 values on a day for HCG (pg/ml)	2678.82 ±719.70 pg/ml	1702.90±700
*Protocol*		
Protocol with antagonists	15 (30.0%)	34 (57.6%)
Long protocol with agonists	35 (70.0%)	25 (42.37%)
Total gonadotropin dose	1759.78±395.09	2325.13±767.87

Statistically significant difference between the two categories occurs in a number of obtained oocytes, E2 values, total gonadotropin dose (p<0.000), as well as in protocols (p=0.014).

**Table III-B:** Demographic and clinical characteristics (mean value ± standard deviation or number (%)) – singleton pregnancy.

	OHSS (n=31)	Control group (n=38)
Age (years)	31.44±3.46	34.14±3.17
BMI (kg/m^2^)	22.08±2.95	22.12±2.59
*Infertility cause*		
PCOS	8 (25./8%)	8(21.0%)
Tubal	4 (12.9%)	4(10.5%)
Endometriosis	2 (6.5%)	3(7.9%)
Anovulation	1(3.2%)	
Male	3 (9.7%)	13 (34.2%)
Unexplained	13 (41.9%)	10 (26.3%)
Number of obtained oocytes	12.31±3.83	8.3±2.71
E2 values on a day for HCG (pg/ml)	2692.70±788.23	1815.49±794.97
*Protocol*		
Protocol with antagonists	10 (32.2%)	25(59.5%)
Long protocol with agonists	21 (67.7%)	17(40.5%)
Total gonadotropin dose	1869±420.63	2442.95±765.95

As in the previous case, significant difference occurs in a number of obtained oocytes, E2 values, total gonadotropin dose (p<0.001).

**Table III-C:** Demographic and clinical characteristics (mean value ± standard deviation or number (%)) – twin pregnancy.

	OHSS (n=19)	Control group (n=21)
Age (years)	34.21±4.10	32.52±4.04
BMI (kg/m^2^)	23.13	21.51±2.11
*Infertility cause*		
PCOS	6 (31.6%)	3 (14.3%)
Tubal	3 (15.8%)	0
Endometriosis	3 (15.8%)	3 (14.3%)
Anovulation	0	0
Male	5 (26.3%)	6 (28.6%)
Unexplained	2 (10.5%)	9 (42.9%)
Number of obtrained oocytes	11.84±3.54	7.90±2.20
E2 values on a day for HCG (pg/ml)	2655.43±606.50	1771.19±504.34
*Protocol*		
Antagonists	5 (26.3%)	10(47.6%)
Agonists	14 (73.7%)	11(52.4%)
Total gonadotropin dose	1817.50±406.37	2532.47±756.94

Statistically significant difference occurs in a number of obtained oocytes (p=0.002), in E2 values, total gonadotropin dose (p≤0.001) as well as in protocols (p=0.010).

## DISCUSSION

The effect of severe form of OHSS on fetal-maternal complications in pregnancy is still insufficiently examined.^12^ The conclusions of various authors often did not coincide, but the most common mentioned complications in pregnancy are hypertensive disease and a higher rate of preterm birth.^13^-^15^ In this study, premature birth was significantly more common in patients with severe form of OHSS than in the control group. Birth weight was also lower in comparison to the control group. Factors, which are not direct results of OHSS, but may have impact on poor perinatology outcomes additionally, complicate interpretation of the results. Further, infertility and assisted reproductive technologies, as two independent factors might have some impact on poor outcomes of such pregnancies.^16^ In terms of pathophysiologal mechanisms responsible for preterm birth a number of cytokines that are produced and which are part of the pathophysiological events during the development of OHSS could also be important. In pathogenesis of OHSS, as well as in inflammatory cascade leading to a preterm birth, proinflammatory cytokines such as interleukins 2, 6 and 8 and VEGF could be of importance.^4^,^17^ A severe form of hyperstimulation followed by an increased permeability of blood vessels with consequent hemoconcentration and hemodynamic instability could have had an impact on the placentation process. The placentation process could also have an impact on possible intrauterine growth restriction. In our study, there was no statistical difference in terms of intrauterine growth restriction of a fetus, but there was statistical difference in weights of newborns which were significantly lower in the study group. Preterm birth was more frequent within OHSS group, which was also the case in the study conducted by Courbiere et al. while Bastek et al. suggested that there is a relation between inflammation, placental dysfunction and preterm birth.^5^,^18^ In studies conducted by Haas et al. in addition to the study group, as a whole with a severe form of OHSS, they also observed patients in which paracentesis, i.e. drainage of ascites or pleural effusion was performed, namely these were the patients with the most severe, life-threatening form of OHSS where the most oocytes were obtained among the others. This group of patients was not in a greater risk for other perinatal complications except for higher frequency of preterm birth in comparison to the control group, which is in line with our findings.^13^,^14^

The obtained results suggest that E2 and the number of aspirated oocytes can be considered relatively reliable predictive factors for the development of OHSS. A large number of aspirated oocytes have been confirmed by other authors as a good predictive factor for the development of OHSS. Estradiol values in some studies were significantly higher in examined patients, while other studies did not confirm its predicative role.^19^,^20^ Gonadotropin dose was significantly lower in the OHSS group. Higher levels of estradiol with lower doses of gonadotropin could explain the predisposition of the patient for the development of OHSS. The role of antagonists in the prevention of OHSS development coincides with our results. Namely, the use of antagonists was statistically significantly higher in the control group. Although use of GnRH antagonists in some controlled clinical trials showed that the frequency of the severe form of OHSS is lower than in case when agonists are used, one cannot exclude a probability that such results are a consequence of possibility that agonists are often used instead of hCG in protocols with antagonists as ovulation triggers, which may itself reduce incidence of OHSS.^21^ However, patient’s predisposition for developing OHSS is still considered as the most probable factor which is also in line with our results.

### Limitations of the study

Since most of the study is retrospective, and less prospective, some data were not available. Frequency of infections within the observed groups as a potential cause of preterm birth was not known. Further, anamnestic data on possible interventions on the cervix were not taken into account. However, as a standard procedure, cervicometry is done in the second trimester, between 20th and 24th week of gestation so patients with cervical insufficiency, i.e. having length less than 25 mm were not taken for study. For investigating infertility, all patients suspected for anomalies of uterus are subjected to 3D ultrasonography examination. If some anomaly is found, patients start with IVF procedure only after it is corrected.

The lack of large prospective studies indicates the need for further research to obtain final and more precise conclusions about the independent and direct impact of severe form of OHSS on the onset of preterm birth.

### Author`s Contribution

**AD and SR** conceived, designed and did statistical analysis.

**AD and SM** did data collection and manuscript writing.

**SR** did review final approval of manuscript.
